# A Case of Rupture of the Uterus, with Artificial Anus at the Point of Rupture

**Published:** 1844-12

**Authors:** William N. Morgan

**Affiliations:** Germantown, Tenn.


Art II.—*A Case of Rupture of the Uterus, with Artificial Anus at the point of rupture.* By William N. Morgan, M.D., of Germantown, Tenn.

Caroline, a colored woman, was attacked with severe labor pains, and all the concomitant symptoms of child-bearing, on the 20th December, 1841. The pains effected a speedy rupture of the membranes, and a consequent discharge of the liquor amnii at the onset of her labor.

She remained in this situation, with excruciating pains, for something like 26 hours, attended by a midwife. At the end of this time a physician was called in, who proceeded to make an examination *per vaginam*, and found the waters discharged, and the child low down in the pelvis, firmly grasped by the uterus, and the pains still very severe.

The presentation was one of the shoulder, the arm having descended. The first step of the medical attendant was to give her an anodyne and bleed her freely from the arm, under which the pains diminished in severity, and he was able to proceed to turn and deliver by the feet, though with much difficulty.

The woman after delivery seemed as well as usual, (for this was her sixth labor) except that she was troubled with costiveness, which had existed some seven or eight days previous to parturition.

The above history I received from the physician and from the mistress of the patient.

Thirty-six hours after the delivery of the woman, I was called in haste to see her, and found her laboring under obstinate constipation of the bowels, though she had taken 9 or 10 ounces of castor oil, and stimulating enemas had been freely used. With this, great soreness of the abdomen existed, accompanied by extreme distension from the lower portion of the sternum to the pubis; her tongue was furred; pulse 120 a minute. The purgatives employed having accomplished nothing, I ordered ol. ricini ℥ij., ol. tiglii gtt.ii., to be administered forthwith; and a blister plaster to be applied to the abdomen.

By persevering in the use of these cathartics, calomel, gamboge, &c., aided by enemas, her bowels were evacuated in four days, after having been inactive ten or twelve.

From this period, there was an abatement of all the alarming symptoms, attended with an evident improvement of the system in general, though the uterus remained above its natural situation, much hardened, rather enlarged, and extremely painful on pressure. She continued to improve slowly for about ten days. At the expiration of this time, she was suddenly and violently attacked with uterine hæmorrhage, attended with great relaxation of the womb, and prostration of the vital energies.

I was again called in haste to see her, and found her in the condition above described. The hæmorrhage yielded to the use of the acetate of lead, opium and ipecacuan, assisted by frictions over the abdomen, and also by astringent injections *per vaginam.*

So soon as she recovered from the prostrating effects of hæmorrhage, she again began to improve as before, with regular discharges from her bowels, and a slight inclination to diarrhœa, which, however, was easily controlled by the use of calomel and opium. She remained in this condition for seven days after the flooding ceased, making about eighteen from the period of her confinement, when I was greatly surprised to learn that she was discharging fæcal matter through the vagina, and none in the natural way. I immediately proceeded to an examination, expecting to find a communication between the rectum and vagina; but finding no rupture there, pushed my examination further. About the left fundus of the uterus the patient had complained much of soreness, and on pushing the fingers into that region a large collection of fæcal matter was discovered. A syringe being introduced, several ounces of fluid fæces were removed. Dr. John Evans being present, satisfied himself by an examination of the existence of such a state of things. She continued to void her fæces involuntarily by the vagina thirty days, during which time she experienced rapid emaciation. About the last of January a favorable change took place. The evacuation of fæcal matter through the uterus ceased, and she began gradually to improve in health and strength, suffering occasionally from pain about the point of the rupture, and being troubled with œdema of the face, feet, and hands.

It is now about three years since this case occurred. The patient has enjoyed tolerable health since the time above mentioned, with the exception of occasional colic pains in the region of the colon, irregular menstruation, and slight diarrhœa. At times she complains of soreness to the left of the umbilicus, at the point where the rupture occurred. She has had frequent communications with her husband, and enjoys the connexion as before, but has not conceived, although she bred rapidly before this accident occurred.

On her recovery from this state of things, when not under the influence of cathartics, her fæces are of the natural shape and color; the action of her bowels is easily induced, and without more pain than is common under the ordinary operation of cathartics. During the time that her fæces were passing *per vaginam*, the only medicines that were given were tonics, with an occasional dose of morphine.

*Remarks.*—The rupture in this case must have occurred during labor, though the intestine could not have passed into the opening until after relaxation of the uterus took place. This must have occurred at the time of the hæmorrhage, for it was not until after the flooding came on (five or six days) that I ascertained that this state of things existed, and this was about the period required for sloughing to take place. At no time were there any symptoms indicating the existence of stricture except at the beginning of the case, as already stated.

October 27, 1844.

